# A Rare Case of Adult Acute Ileocecal Ischemia Related to Henoch-Schönlein Purpura: MDCT Findings

**DOI:** 10.5334/jbr-btr.932

**Published:** 2016-02-17

**Authors:** Bruno Coulier, Luc Montfort, Vincent Cloots, Muriel Parent

**Affiliations:** 1 Clinique Saint-Luc, Bouge, Belgium, BE; 2 Clinique St Luc, Bouge 5004 (Namur), BE; 3 Institute of Pathology and Genetics, Loverval, Belgium, BE

**Keywords:** Henoch-Schönlein purpura, Abdominal pain, Gastrointestinal bleeding, Mesenteric vasculitis, Abdomen, CT

## Abstract

Henoch-Schönlein purpura (HSP) is a form of immune complex-mediated leukocytoclastic vasculitis involving the skin and other organs. It primarily affects children. The occurrence of HSP in adults is rare, and gastrointestinal (GI) involvement is one of its most common clinical manifestations. The GI symptoms are caused by hemorrhage and edema within the bowel and wall mesentery. Complete recovery usually occurs, and life-threatening complications are rare. We report a typical case of GI involvement of the ileocecal area diagnosed with multidetector computed tomography (MDCT) and confirmed by skin biopsy.

## Introduction

Henoch-Schönlein Purpura (HSP) is a form of immune complex-mediated leukocytoclastic vasculitis involving the skin and other organs. It primarily affects children, and the occurrence in adults is rare and only sporadically reported. Gastrointestinal (GI) involvement is one of the most common manifestations of HSP in adults. The common symptoms are abdominal pain, colorectal bleed or occult blood loss, vomiting, and diarrhea. These symptoms are caused by hemorrhage and edema within the bowel and wall mesentery. Complete recovery usually occurs, and serious complications leading to surgery are rare. We report a typical case of GI involvement of the ileocecal area diagnosed with multidetector computed tomography (MDCT) and confirmed by skin biopsy.

## Case Report

A 62-year-old man presented to the emergency department with complaints of colicky abdominal pain and active rectorrhagy. Two days before, these symptoms had been preceded by the apparition of diffuse purpuric spots on the lower limbs and the forearms. One week before, the patient had experienced a viral episode of the nasopharyngeal sphere. At admission, the patient was apyretic and articular symptoms were absent. Physical examination revealed a compressible and painless abdomen. Peristalsis was preserved, and laboratory blood tests were normal except a mildly elevated IgA level. There were only subtle urinary signs of glomerular damage with microscopic hematuria, mild proteinuria, and mixed cellular urinary casts.

Rectosigmoidoscopy was normal except for rare bloodstains. Gastroscopy revealed diffuse duodenal small necrotic ulcers.

**Figure 1:**
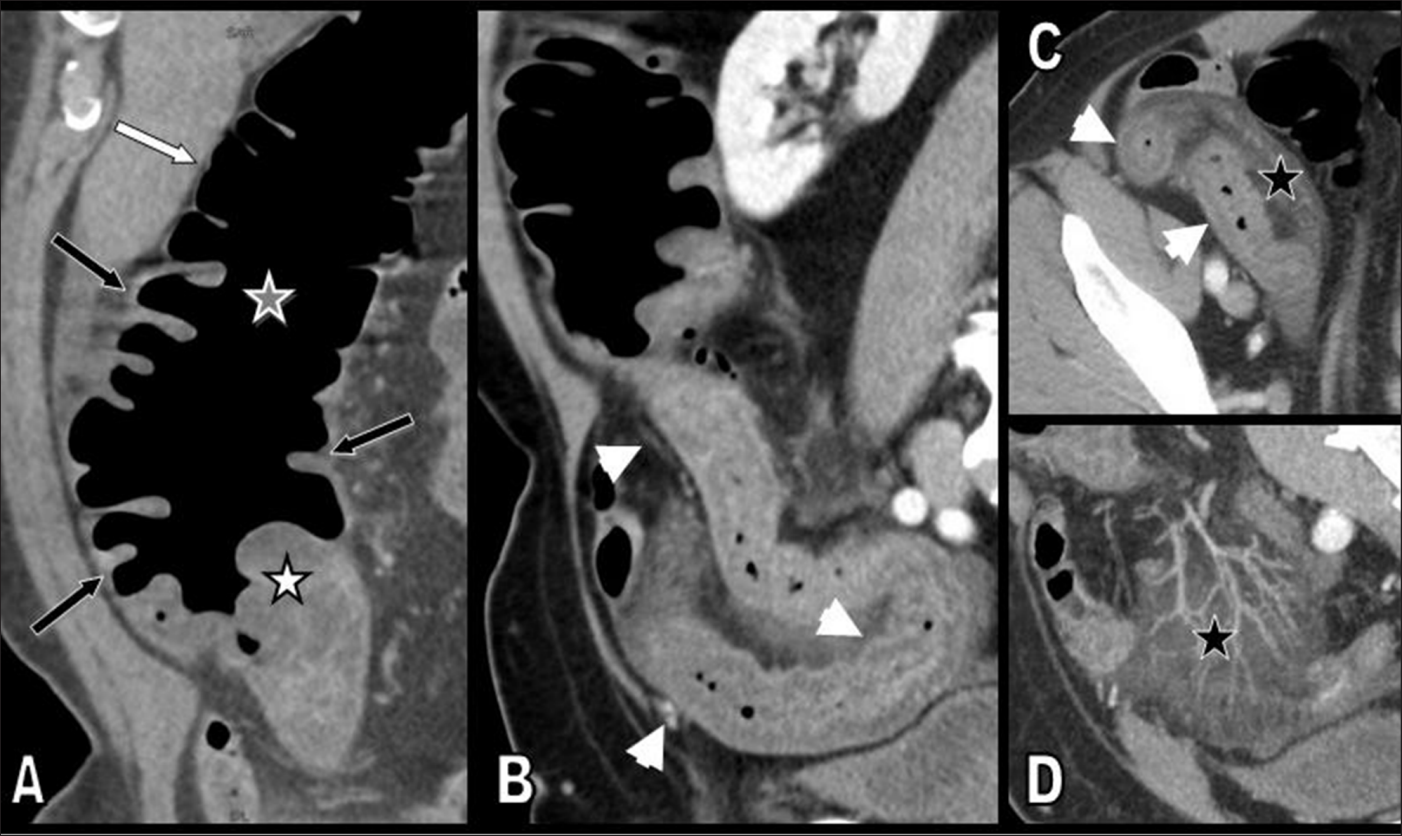
Coronal oblique multiplanar reconstruction (MPR) (A), sagital oblique MPR (B), axial view (C), and coronal oblique maximal intensity projection (MIP) (D) of the level of the right iliac fossa illustrate a circumferential and continuous segmental thickening of the distal ileum (small white arrows), cecum, and proximal ascending colon (black arrows). The limit between the affected and the normal bowel is extremely sharp, especially at the level of the ascending colon (grey star). The target sign or stratified pattern is continuously present, with mucosal hyperemia contrasting with hypodensity of the edematous submucosa. Edema is maximal at the level of the Bauhin’s valvule, which appears very turgescent (white star). Massive and sharply delimitated fat stranding and edema is visible in the neighboring ileo-cecal mesentery (black star). Ascite is also present in the abdominal cavity (not illustrated).

**Figure 2:**
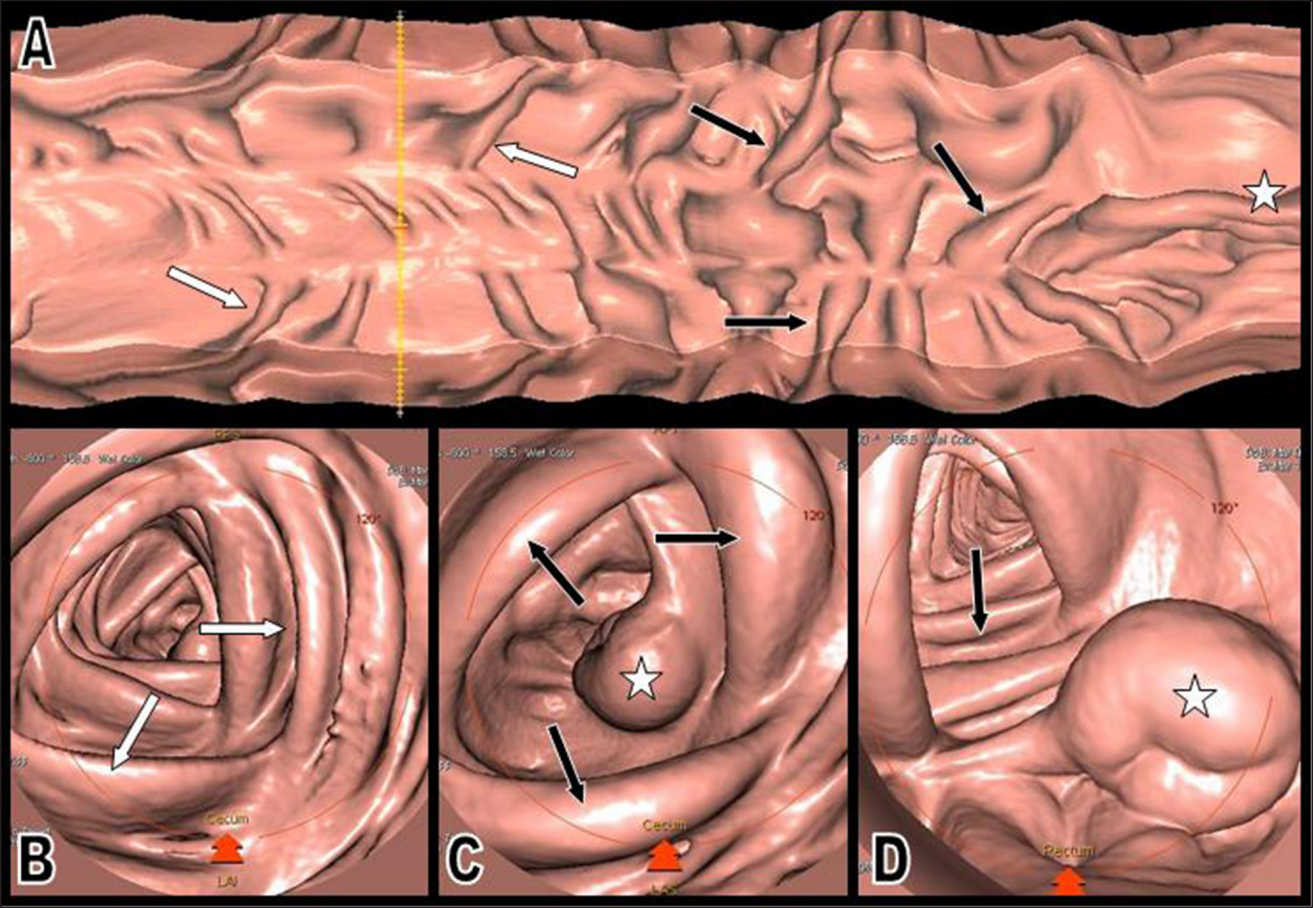
Virtual endoscopic views (A–D) of the ascending colon clearly illustrate the turgescent edema of the Bauhin’s valvule (white star) and of the proximal colonic haustrations (black arrows). The more distal haustrations appear normal (white arrows).

**Figure 3:**
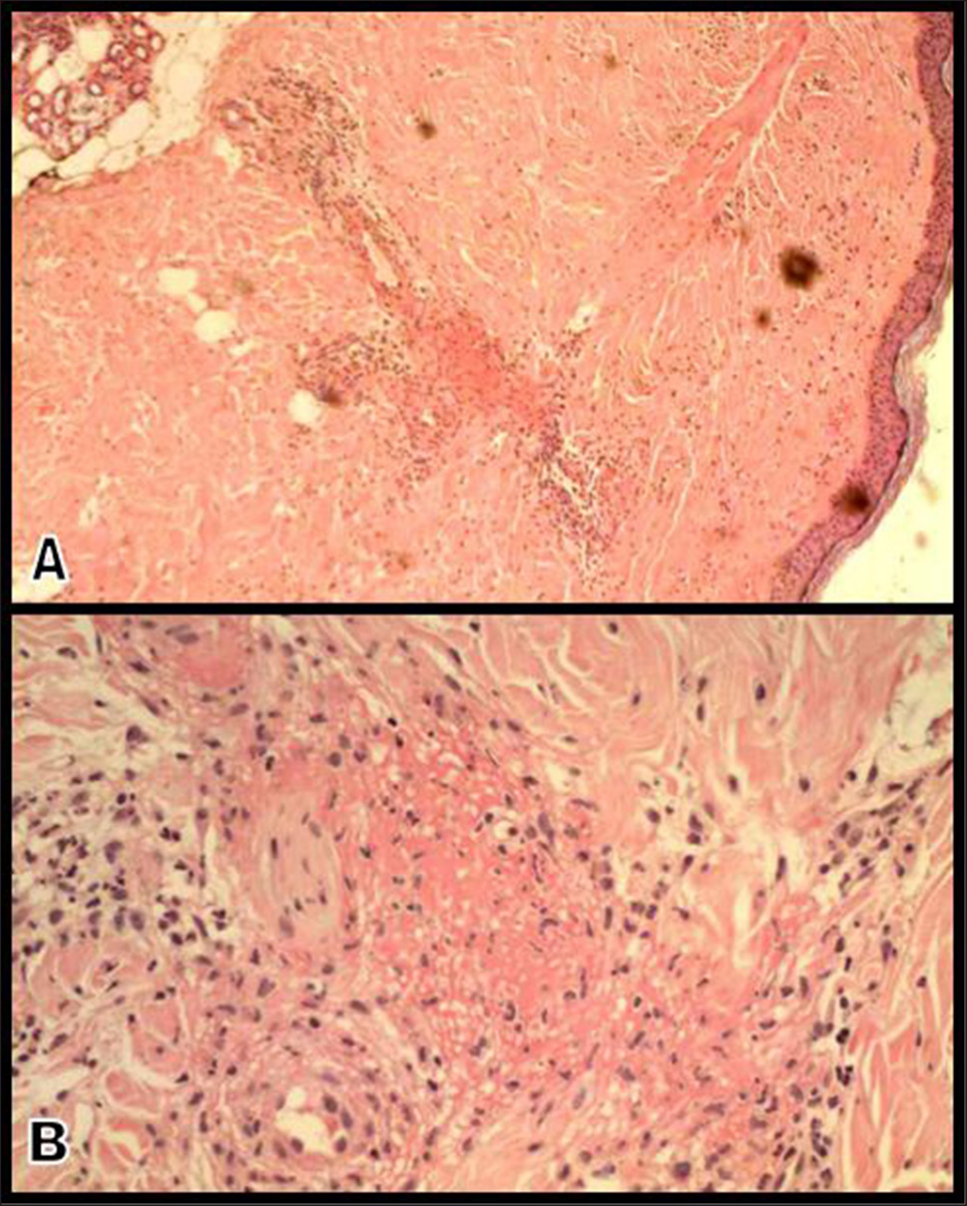
Photomicrographs of skin biopsy specimen – Hematoxylin and Eosin, × 100 (figure A) and × 400 (figure B) – show typical intradermal leucocytoclastic vasculitis. Inflammatory neutrophilic infiltrate and fibrinoid necrosis surround the blood vessels with karyorrhexis (figure A). The endothelial cells have disappeared in fibrinoid necrosis with extravasation of red blood cells (figure B).

Contrast-enhanced multidetector-row CT (Figure 1) demonstrated homogenous circumferential bowel thickening of a rather long segment of the ileum terminal. The typical “target sign” (or stratified pattern) was present with hypodense edema of the submucosa contrasting with hyperemia or hyperperfusion of the mucosa. Engorgement of the ileocecal mesenteric vessels with typical comb sign was also present. Hypodense thickening of the cecum and proximal ascending colonic wall was also clearly present, and the demarcation between the thickened and normal colonic wall appeared very sharp on conventional CT views (Figure 1) and virtual endoscopic views (Figure 2). A small amount of ascite was also found in the pelvic floor and in the perihepatic space. Skin biopsy revealed typical signs of leukocytoclastic vasculitis (Figure 3).

The final diagnosis of Henoch-Schönlein purpura with gastrointestinal involvement was proposed, and the patient was successfully treated with an association of corticosteroids and cyclophosphamide. Gastrointestinal symptoms resolved within two days, and there was no recurrence of symptoms.

## Discussion

Henoch-Scönlein purpura (HSP) is a small vessel leukocytoclastic vasculitis with the deposition of immune complexes containing IgA. It is clinically characterized by a clinical tetrad comprising palpable purpura, arthritis, and renal and gastrointestinal involvement that may occur in successive episodes. It is the most common acute vascular illness affecting children with a reported mean age of 5.9 years, a peak occurrence at 3–5 years, and an incidence of approximately 15 cases/100,000 children per year. Nevertheless, HSP is generally a self-limiting process in children with an excellent prognosis [1,2,3,4,5,6].

The etiology of HSP remains unknown. Various antigenic stimuli have been proposed, including a broad spectrum of bacteria, viruses, or allergens, including vaccines, tumor antigens, autoimmune diseases, drugs, foods, and exposure to cold [5,7]. The clinical manifestations are caused by deposition of IgA immune complexes on the intima of small blood vessels, leading to complement activation, leukocyte recruitment, and destruction of endothelial cells [7].

Histopathologically immune complex deposits induce necrosis of the walls of small and medium-sized arteries with infiltration of the tissue by neutrophils and the deposition of nuclear fragments, a process called “leukocytoclastic vasculitis” [8].

HSP is rather uncommon in adults, with sporadic reports in patients up to 86 years old and occurrence of 3.4 to 14.3 cases per million [1,5]. Although HSP has been extensively studied in children, much less is known about its natural history in adults which display higher morbidity and mortality. The long-term prognosis merely depends on the severity of renal involvement – ranging from microscopic hematuria to a full nephrotic syndrome – which may result in renal insufficiency in about 30 per cent of adults [2,4,5]. Cases of deaths have been reported in adult HSP with severe gastrointestinal (GI) involvement [4,9].

GI involvement is one of the most common manifestations of HSP in adults, with a reported incidence of 56–85 per cent [1,3,4]. The common symptoms are abdominal pain (86%), massive colorectal bleed (20%), occult blood loss (66%), vomiting (40%), and diarrhea (20%). The symptoms are caused by hemorrhage and edema within the bowel and wall mesentery [3,7]. Serious complications related to GI involvement include ileoileal intussusceptions, infarction, perforation, fistula, massive bleeding, protein-losing enteropathy, hemorrhagic ascites, and pancreatitis [3,4,9].

Cutaneous purpura is necessary for the diagnosis of HSP, although there have been reports of GI symptoms preceding cutaneous purpura by 2–14 days in 8 per cent of cases [1,5]. As in the reported case, an elevated IgA level occurs in about 60 per cent of cases [5].

The small intestine is the most common site involved with a predilection for the terminal ileum (60%) and the second portion of the duodenum (53%). The rectum (80%) is the most frequently affected segment of the lower GI tract [3,8]. Endoscopic findings include mucosal congestion, redness, petechiae, multiple ulcers, and hemorrhagic erosions [3,8]. In most cases, the GI manifestations are rarely severe enough to require surgical intervention [10].

Given the potential severity and sometimes fatal outcome of GI involvement of HSP in adults, a prompt and early diagnosis is crucial, and CT plays an essential role [3].

Vasculitis is an inflammation of blood vessel walls, causing alteration of the blood flow and secondary damage to the affected organ [11]. All types of vasculitis can cause local or diffuse pathologic change in the gastrointestinal tract [11]. The great variety of damage includes ulcers, submucosal edema, hemorrhage, paralytic ileus, mesenteric ischemia, and bowel obstruction or perforation.

Vasculitis is classified as primary or secondary. Primary vasculitis is classified into large-vessel vasculitis (giant-cell arteritis and Takayasu’s arteritis); medium-sized-vessel vasculitis (comprising polyarteritis nodosa and Kawasaki’s disease); and small-vessel vasculitis (a larger group comprising Wegener’s granulomatosis, Churg-strauss syndrome, microscopic polyangitis, Henoch-Schönlein purpura, essential cryoglobuliemia, and cutaneous leukocytoclastic vasculitis). Secondary vasculitis essentially refers to small-vessels vasculitis associated to connective tissue diseases, infectious diseases, drugs, and various paraneoplastic vasculitis [11]. The frequency of GI involvement in large-vessel vasculitis is rather rare. In medium-sized vessel vasculitis, polyarteritis nodosa may dramatically affect two-thirds of patients, and the clinical course is often dramatic. In small-vessel vasculitis, the GI tract is essentially affected in Churg Strauss syndrome, but also frequently in Henoch-Schönlein purpura, Systemic lupus erythematous, and Behcet’s disease.

Many drugs and infectious agents can cause pathological features being undistinguishable from small-vessel primary vasculitis. Vasculitis or connective tissue diseases and malignancy are related and vice versa, malignancies occurring more frequently in the course of vasculitis and vasculitis occurring in the course of malignancy. A screening for occult or misdiagnosed malignancy is thus necessary in patients suffering from vasculitis [11].

Nevertheless, vasculitis remains a rather rare cause of GI ischemia (with the highest prevalence in polyarteritis nodosa), and consequently the distinction between GI ischemia related to vasculitis and more common causes of mesenteric ischemia may be difficult on the basis of radiological signs alone [12]. The diagnosis should be evocated when mesenteric ischemia produces in rather young patients; involves unusual sites such as the stomach, the duodenum, and the rectum; or does not remain confined to a single territory and is accompanied by other systemic clinical symptoms [12].

Thickening of the bowel wall is the most common but least specific CT sign of bowel ischemia [11]. The extent of involvement, degree of thickness, and pattern of attenuation of the ischemic bowel depend on three main factors: the pathogenesis of the ischemia (arterial-occlusive, veno-occlusive, or hypoperfusion); the severity of the ischemia (transient ischemia of the mucosa or submucosa versus transmural bowel wall necrosis); and eventual superimposed hemorrhage or infection. Nevertheless, the ischemic bowel wall may also appear paper thin, particularly in cases of acute arterial occlusion.

The most characteristic CT findings of gastrointestinal involvement in vasculitis include bowel thickening with the target sign (or stratified pattern) and engorgement of mesenteric vessels with comb sign [11]. The stratified pattern result from edema of the submucosa and hyperhemia or hyperperfusion of the mucosa or muscularis propria [12].

The empirical use of corticosteroids in the treatment of HSP was introduced in 1950s, and abundant clinical experience supports the value [1] of high-dose corticosteroids in ameliorating the GI symptoms. Other immunosuppressive agents such as cyclophosphamide, azathioprine, and mycophenolate motefil have also been used [3].

## Competing Interests

The authors declare that they have no competing interests.
